# Combination of Hypotonic Lysis and Application of Detergent for Isolation of Polyhydroxyalkanoates from Extremophiles

**DOI:** 10.3390/polym14091761

**Published:** 2022-04-26

**Authors:** Ivana Novackova, Xenie Kourilova, Katerina Mrazova, Petr Sedlacek, Michal Kalina, Vladislav Krzyzanek, Martin Koller, Stanislav Obruca

**Affiliations:** 1 Faculty of Chemistry, Brno University of Technology, Purkynova 118, 612 00 Brno, Czech Republic; xcnovackova@fch.vut.cz (I.N.); xckourilovax@fch.vut.cz (X.K.); sedlacek-p@fch.vut.cz (P.S.); kalina-m@fch.vut.cz (M.K.); 2 Institute of Scientific Instruments of the Czech Academy of Sciences, v.v.i., Kralovopolska 147, 612 64 Brno, Czech Republic; mrazova@isibrno.cz (K.M.); krzyzanek@isibrno.cz (V.K.); 3 Research Management and Service, c/o Institute of Chemistry, NAWI Graz, University of Graz, Heinrichstrasse 28/IV, 8010 Graz, Austria; martin.koller@uni-graz.at; 4 ARENA—Arbeitsgemeinschaft für Ressourcenschonende & Nachhaltige Technologien, Inffeldgasse 21b, 8010 Graz, Austria

**Keywords:** polyhydroxyalkanoate (PHA), sodium dodecyl sulfate (SDS), PHA isolation, extremophiles, halophiles, thermophiles, *Halomonas halophila*, *Schlegelella thermodepolymerans*

## Abstract

Production of polyhydroxyalkanoates (PHA), microbial biopolyesters, employing extremophilic microorganisms is a very promising concept relying on robustness of such organisms against microbial contamination, which provides numerous economic and technological benefits. In this work, we took advantage of the natural susceptibility of halophilic and thermophilic PHA producers to hypotonic lysis and we developed a simple and robust approach enabling effective isolation of PHA materials from microbial cells. The method is based on the exposition of microbial cells to hypotonic conditions induced by the diluted solution of sodium dodecyl sulfate (SDS) at elevated temperatures. Such conditions lead to disruption of the cells and release of PHA granules. Moreover, SDS, apart from its cell-disruptive function, also solubilizes hydrophobic components, which would otherwise contaminate PHA materials. The purity of obtained materials, as well as the yields of recovery, reach high values (values of purity higher than 99 wt.%, yields close to 1). Furthermore, we also focused on the removal of SDS from wastewater. The simple, inexpensive, and safe technique is based on the precipitation of SDS in the presence of KCl. The precipitate can be simply removed by decantation or centrifugation. Moreover, there is also the possibility to regenerate the SDS, which would substantially improve the economic feasibility of the process.

## 1. Introduction

Polyhydroxyalkanoates (PHAs) are microbial storage polyesters, which are accumulated by various prokaryotic microorganisms. Apart from their primary storage function for carbon, energy, and reduction equivalents, PHAs also significantly enhance the stress robustness of microorganisms and accumulation of them is probably a part of adaptation to some extreme conditions such as high salinity [1]. PHAs are, therefore, widespread metabolites among extremophilic prokaryotes [2]. Moreover, they represent a very promising ecologically friendly alternative to traditional petrochemical polymers–PHAs are completely biodegradable and compostable materials, which can be produced from totally renewable resources including also waste products stemming from food production and other industries. Their properties and application fields are very similar to petrochemical plastics such as polypropylene [3].

Nevertheless, the main problem prohibitive for large-scale production of PHAs is their high production cost. There are several strategies how to overcome this limitation. One of them is the employment of extremophiles as PHA-producing chassis. The main advantage of extremophiles is their capability of growth and production of various metabolites including but not limited to PHA under conditions, which reduce or even eliminate the risk of contamination of the process by common mesophilic microflora, e.g., high or low temperature, high salinity, extreme pH-values, etc. Due to their robustness against unfavorable microbial contamination, which can destroy whole fermentation batches, biotechnological processes using extremophilic microorganisms can be operated under reduced requirements for sterility, or eventually even completely without sterilization of the cultivation media or equipment. Consequently, it has a significantly positive impact on the energy and economic balance of these processes. Moreover, high tolerance of the process against contamination allows running the cultivation process in highly productive continuous or semi-continuous mode. Therefore, employing extremophilic microorganisms in biotechnology is nowadays a modern trend, which is called the concept of industrial biotechnology of the next-generation (“Next-Generation Industrial Biotechnology”, NGIB) [4].

In this context, the employment of extremophilic microorganisms can significantly reduce the price of PHA. It can take advantage of the ability of numerous extremophiles to produce PHA natively. Predominantly many halophilic microorganisms (microorganisms adapted to the high salinity of environments) belong among promising PHA producers–PHA production ability was described for several representatives of genus *Halomonas* such as *Halomonas boliviensis* [5], *Halomonas neptunia* and *Halomonas hydrothermalis* [6], *Halomonas bluephagenesis* [7], *Halomonas halophila* [8], or extremely halophilic representatives of the Archaea domain, such as *Haloferax mediterranei* [9]. As mentioned above, the accumulation of PHA is a part of the adaptation strategy of halophiles to hyperosmotic environment; however, the main adaptation strategy is usually the accumulation of either organic (osmolytes) or inorganic (K^+^ ions) substances, which compensate for the osmotic pressure of the surrounding, as observed especially for extreme haloarchea [10].

Instead of halophiles, the production of PHAs was also observed in a restricted number of thermophilic bacteria, which are adapted to high temperature condions [2]. The most studied strains belong to the genera *Caldimonas* [11], *Tepidimonas* [12], in addition to the very promising PHA producer *Schlegelella thermodepolymerans*, which showed expedient potential for PHA production using lignocellulose-based substrates with high xylose content [13]. For thermophiles, PHA most likely does not directly take the role in the adaptation to high temperature, since the adaptation is usually connected with changes of protein structure, enhancement of the activity of chaperones and other heat-shock proteins, and also accumulation of organic compatible molecules similarly to halophilic prokaryotes [14].

Apart from the microbial biosynthesis of PHA, also the expenses associated with the isolation of PHA, an intracellular product, from microbial biomass contribute to the high cost of PHA. Extraction of the polymer from the microbial cells is often performed by organic solvents; however, considering limited polymer solubility, the extraction is limited to selected, predominantly chlorinated, solvents such as chloroform or dichloromethane. Alternatively, compounds typically described as “PHA anti-solvents”, such as acetone, can be employed for PHA extraction under conditions of highly elevated temperature (120 °C) and pressure (7 bar) [15]. Nevertheless, the approaches based on organic solvents are technologically difficult, and the use of toxic and hazardous solvents eliminates the positive ecological impact of final materials [16]. The other possibility is the utilization of supercritical CO_2_ for PHA extraction. Despite a very positive ecological connotation, due to the low solubility of PHA in supercritical CO_2_ and the need for additional compounds acting as solubility mediators (“modifiers”), such as methanol, the process shows low efficiency [17,18]. Also, an alternative PHA isolation approach based on the removal of other components of biomass can be used, when PHA granules remain in the intact, solid state and can be further separated by centrifugation or filtration. Form an economical viewpoint, this approach makes definitely sense: Considering the fact that the PHA fraction in biomass can exceed 90 wt.%, it appears cumbersome to recover these 90 wt.% instead of simply removing the minor (10%) fraction of biomass. Indeed, many reagents and strategies can be used to remove other cell components instead of PHA. For instance, in literature the use of hydrolytic enzymes (predominantly commercially available proteases–Protease L330, Esparase, Alcalase, Neutrase, Allprotease, etc.) is described [19], but in practice, these approaches are limited by the high cost of enzymes. An alternative way is the digestion of biomass using various chemical reagents such as sodium hydroxide [20], sodium hypochlorite [21], EDTA, and detergents such as SDS [22] or ammonium laurate [23]. Individual approaches can be combined as was mentioned in Kathiraser et al., who used enzymes together with detergents and EDTA for PHA isolation [24].

Hypotonic lysis is an additional tool that can be used for the simple disruption of microbial cells. This approach is based on the exposition of cells to surrounding with significantly lower osmotic strength than osmolarity of cultivation medium. During sudden exposition to a hypotonic environment, water begins to penetrate into cells, which leads to disruption of their integrity, rupture of cells, and release of the intracellular cell content. This hypoosmotic cell lysis approach was described predominantly for extreme halophilic prokaryotes such as *Hfx. mediterrenei*, which are cultivated in media with salt contents of more than 200 g/L and are, therefore, extremely sensitive to hypotonic treatment. During exposure to distilled water, cells disruption and release of PHA granules into the solution occur immediately. After centrifugation, there are two phases generated—the bottom pinkish phase represents cell mass residues (colorized by carotenoid pigments), while the upper white phase consists of PHA granules [25]. However, the purity of PHA isolated in the described way is not too high due to the PHA granules being covered by a proteinaceous membrane; therefore, the following purification using for instance hypochlorite, H_2_O_2_, or other reagents is usually necessary [8,26].

In this work, we decided to investigate the possibility of utilization of hypotonic lysis to isolate PHA from moderately extremophilic microorganisms—halophilic *Halomonas halophila* and also thermophilic *Schlegelella thermodepolymerans.* We hypothesized that both microorganisms could be partially sensitive to hypotonic lysis due to the accumulation of compatible solutes as a strategy of their adaptation to extreme conditions. To further support the process of cell disruption and increase the purity of the isolated materials, we have also introduced a low concentration of 10 g/L of sodium dodecyl sulfate (SDS). Since SDS might reduce the positive environmental impact of the process by serious contamination of wastewater, we introduced a step in which SDS leftovers after PHA isolation are precipitated with KCl and can be easily removed from wastewater and potentially even regenerated for repeated use.

## 2. Materials and Methods

### 2.1. Cultivation of Microorganisms

For experimental work, two extremophilic strains were used, the moderately halophilic strain *Halomonas halophila* (CCM 3662) obtained from Czech Collection of Microorganisms, Brno, Czech Republic, and the moderately thermophilic strain *Schlegelella thermodepolymerans* (DSM 15344) purchased from Leibnitz Institute DSMZ—German Collection of Microorganisms and Cell Cultures, Braunschweig, Germany.

The first cultivation step, preparation of inoculum, was performed in 100 mL Erlenmeyer flasks with 50 mL of media. Inoculum culture of *H. halophila* was prepared in a medium consisting of yeast extract (3 g/L), peptone (15 g/L), glucose (1 g/L), and sodium chloride (66 g/L) during 24 h in 30 °C, permanent shaking 180 rpm. Inoculum of *S. thermodepolymerans* was prepared in 25 g/L Nutrient Broth medium (10 g/L peptone, 10 g/L beef extract and 5 g/L NaCl) during 24 h in 50 °C, permanent shaking 180 rpm.

After 24 h lasting cultivation, mineral salts media were inoculated by 10% (*v*/*v*) of inoculum, cultivations were performed in 250 mL Erlenmeyer flasks with 100 mL of medium. The basic mineral salt medium for *H. halophila* cultivation consisted of (NH_4_)_2_SO_4_ (3.0 g/L), KH_2_PO_4_ (1.02 g/L), Na_2_HPO_4_·12H_2_O (11.1 g/L), MgSO_4_·7H_2_O (0.2 g/L), NaCl (66 g/L), glucose (20 g/L) and 1mL/L of microelements solution (FeCl_3_ (9.7 g/L), CaCl_2_ (7.8 g/L), CuSO_4_ (0.156 g/L),CoCl_2_ (0.119 g/L), NiCl_2_ (0.118 g/L) and CrCl_2_ (0.062 g/L) dissolved in 0.1 M HCl) and 20 g/L of glucose. The mineral salt medium for cultivations of *S. thermodepolymerans* consisted of Na_2_HPO_4_·12 H_2_O (9.0 g/L), KH_2_PO_4_ (1.5 g/L), NH_4_Cl (1.0 g/L), MgSO_4_·7 H_2_O (0.2 g/L), CaCl_2_·2 H_2_O (0.02 g/L), Fe(III)NH_4_citrate (0.0012 g/L), yeast extract (0.5 g/L), 20 g/L xylose and 1 mL/L of microelements solution (EDTA (50.0 g/L), FeCl_3_·6 H_2_O (13.8 g/L), ZnCl_2_ (0.84 g/L), CuCl_2_·2 H_2_O (0.13 g/L), CoCl_2_·6 H_2_O (0.1 g/L), MnCl_2_·6 H_2_O (0.016 g/L), H_3_BO_3_ (0.1 g/L), dissolved in distilled water) (all used chemicals including hydrochloric acid, salts, glucose and xylose were purchased from LachNer, Neratovice, CZE; yeast extract, peptone and Nutrient Broth were purchased from HiMedia, Brno, CZE). Inoculated media were cultivated during 72 h under permanent shaking at 180 rpm at the optimal growth temperature, 30 °C for *H. halophila* and 50 °C for *S. thermodepolymerans*, respectively.

### 2.2. Basic Characterization of Cultures

After 72 h, cultivations were finished. For determination of growth of culture, optical density of cultures was measured using a spectrophotometer (P300, Implen, Munich, Germany) at 630 nm against distilled water as a blank. Gravimetrical determination of cell dry matter (CDM) was performed by centrifugation of 10 mL of culture at 3460× *g*, 5 min (EBA 200, Hettich, Spenge, Germany). The generated supernatant was discarded and the pellet was washed with distilled water and centrifuged again. The pellet was dried to constant weight at 70 °C in drying chamber (IP60, LTE Scientific, Oldham, UK) and then the amount of biomass (g/L) was obtained by weighing on an analytical scale (Pioneer PA224C, Ohaus, Parsippany, NJ, USA). Moreover, the content of PHA in dried biomass was measured by gas chromatography with flame ionization detection (GC-FID; gas chromatograph Trace 1300, Thermo Scientific, column: DB-WAX 30 m by 0.25 mm, Thermo Scientific, Waltham, MA, USA) as reported previously in Brandtl et al. (1988) [27]. All determinations were performed in dublicate.

### 2.3. Isolation of PHA from Bacterial Biomass

Right after cultivation, the isolation of PHA from wet bacterial biomass is performed. Predominantly for halophilic strain *H. halophila*, but also for *S. thermodepolymerans*, a very important strategy is the hypotonic environment, which induces disruption of cells leading to release of the intracellular cell content including PHA granules. The main idea of isolation is based on the use of hypotonic conditions together with SDS (TCI, Zwijndrecht, Belgium) applied in low but still sufficient concentration along with the elevated temperature. These factors should lead to cell disruption and the release of water-insoluble PHA granules. For both microorganisms, we focused at the beginning on the optimization of SDS concentration (in range 1 to 10 g/L), temperature treatment (higher and lower than cultivation temperature), and the ratio of microbial biomass vs. volume of SDS solution.

The first optimized parameter was the most efficient concentration of SDS. The experiment was performed in this way: 10 mL of the culture after cultivation was centrifuged in a plastic test tube with a screw cap (3460× *g*, 10 min), the supernatant was discarded and replaced by prepared aqueous solution of SDS (1; 2.5; 5 and 10 g/L), in replicates for each SDS concentration. Pellets were resuspended and suspensions were incubated in a water bath at 70 °C for 120 min. After incubation, test tubes with suspensions were centrifuged again (3460× *g*, 5 min), supernatants were discarded and preserved for further use, the pellets containing predominantly isolated PHA granules were washed with distilled water and then dried to constant weight at 70 °C. The amount of the product was determined gravimetrically and the purity of obtained PHA was determined by GC-FID as the same as described above.

The second, optimized parameter was the temperature of isolation or combination of different temperatures, i.e., temperature treatment. For both strains, the same treatments were tested: 50 °C (120 min), 70 °C (120 min), 90 °C (120 min) and a combination of cooling-down and heating-up; 4 °C (120 min) followed by 70 °C (120 min). SDS was applied at a concentration of 5 g/L, otherwise, the experiment was performed in the same way as described above for optimization of SDS concentration.

To reduce the amount of detergent (SDS) using a higher biomass amount, the third series of optimization experiments was based on a comparison of the effectivity of isolation with different ratios of initial biomass concentration and 10 mL of aqueous SDS (5 g/L) solution in the most to verify the purity of obtained material and compare product yield. Also, the purpose of this experiment was to verify the robustness of the isolation process concerning different initial biomass concentrations—using a higher ratio of biomass to SDS solution can significantly reduce the cost of the isolation process (amount of SDS per biomass) and also significantly reduce environmental burden caused by SDS in wastewater. In practice, different amounts of biomass were obtained by centrifugation of a larger volume of cell culture when the volume of SDS solution was still 10 mL. However, the approach was the same as previous ones, using optimal SDS solution concentration of 5 g/L for both strains and optimal “heat treatment”: 70 °C for 2 h for *H. halophila* and keeping at temperature 90 °C for 2 h for *S. thermodepolymerans*. All isolation experiments based on long-term exposition to elevated temperatures were performed in a water bath (BL 4/150, WSL, Czestochowa, Poland).

### 2.4. Characterization of Isolated PHA Polymer

In this work, we produced a homopolymer of 3-hydroxybutyrate-poly(3-hydroxybutyrate) (PHB), the most common representative of PHA. Quantification of the polymer in bacterial biomass and determination of purity of the isolated samples were performed by GC-FID as described above.

Further, we used Fourier transform infrared spectroscopy (FT-IR, Nicolet iS50, Thermo Scientific, Waltham, MA, USA) with the built-in single-reflection diamond attenuated total reflectance (ATR) crystal to collect infrared spectra of the original biomass, polymer isolates, and the reference material, commercially available P(3HB) (Biomer, Schwalbach am Taunus, Germany). Each spectrum was collected in the range of 4000–400 cm^−1^ as an average of 16 scans with a resolution of 4 cm^−1^ (data spacing 0.5 cm^−1^).

For determination of molecular weight and its distribution (polydispersity index PDI) of the polymer, we used size-exclusion chromatography with multi-angle light scattering detector (SEC-MALS) (SEC chromatography, column PLgel mixed-C 5 μm, 300 × 7.5 mm, Agilent Technologies, Wilmington, DE, USA; detectors: MALS–DAWN HELEOS II, differential refractometer OPTILAB T-REX, Wyatt Technology, Dernbach, Germany). Using this method, we compared polymers isolated by different approaches, which were the most promising based on results from GC connected with the highest material purity. Approximately 200 mg of sample (biomass, isolated material) was weighed into glass pyrex test tubes, 10 mL of chloroform was added, then test tubes were tightly screwed with caps and during 12 h incubated at 70 °C in a thermoblock. After 12 h, the mixture was filtered through a paper filter on Petri dishes, and chloroform was freely evaporated. Then 1.5 mg of foil was weighed to the vial (volume 4 mL) and 1.5 mL of chloroform (LachNer, Neratovice, CZE) was added. The mixture was incubated at 50 °C in the thermoblock till all foil was dissolved, then the solution was filtered using nylon filters with 0.45 μm pore size into small vials, and the samples were analyzed.

Selected samples were also analyzed by electron microscopy. Bacterial cultures and isolated PHA samples were fixed using the high-pressure freezing method (EM ICE, Leica Microsystems, Wetzlar, Germany). Samples were centrifuged for 3 min at 4000 rpm and the generated pellet was pipetted on the 0.2 mm side of the 6 mm Al carrier type A and closed with the flat side of carrier type B (without using any cryo-protectant) for further processing for observation in the cryogenic scanning electron microscope (cryo-SEM). For freeze-substitution procedure followed by observation in the transmission electron microscope (TEM), samples were frozen in 0.2 mm side of the 3 mm Au carrier type A and closed with the flat side of carrier B. Both of the carriers were pre-treated with 1% aqueous solution of lecithin (Sigma-Aldrich, Darmstadt, Germany) in chloroform. For cryo-SEM, high-pressure-frozen samples were transferred into a cryo vacuum preparation chamber (ACE 600, Leica Microsystems, Wetzlar, Germany), where they underwent freeze-fracture followed by sublimation for 7 min at −95 °C. Samples were then transferred into a scanning electron microscope equipped with a cryo stage (Magellan 400/L, FEI, Hillsboro, OR, USA) and observed using 1 keV electron beam at −120 °C. For TEM, high-pressure-frozen samples were transferred into a freeze substitution unit (AFS2, Leica Microsystems, Wetzlar, Germany) containing a freeze-substitution solution of 1.5% OsO_4_ in acetone. The freeze-substitution protocol was set as previously described in Kourilova et al. [28]. Thus prepared freeze-substitution samples were washed in pure acetone (3 × 15 min) and gradually infiltrated with epoxy resin (Epoxy Embedding Medium, Sigma-Aldrich, Darmstadt, Germany), mixtures with acetone 1:2, 1:1, 2:1 and pure resin for 1 h each. After the final exchange of pure resin, samples were left overnight under vacuum, then embedded in fresh resin and cured at 62 °C heat for 48 h. Cured blocks of samples were cut to ultrathin sections using a diamond knife with cutting angle 45° (Diatome, Nidau, Switzerland) and ultramicrotome (UTC 7, Leica Microsystems, Wetzlar, Germany), and stained using solutions of uranyl acetate and lead citrate. Contrasted ultrathin sections were imaged using a transmission electron microscope (Talos F200C, Thermo Fisher Scientific, Waltham, MA, USA) using a 200 keV beam.

### 2.5. Precipitation of Potassium Dodecyl Sulfate (KDS) and Determination of Residual SDS Concentration

SDS is a water-soluble molecule, whereas the salt potassium dodecyl sulfate (KDS) is water-insoluble. This is the main idea of how to get rid of SDS from wastewater after isolation of PHA from bacterial biomass. A simple addition of KCl to supernatant containing SDS results in the formation of an insoluble precipitate of KDS, which can conveniently be separated by sedimentation or centrifugation. For this purpose, we prepared 4 M aqueous KCl solution and after isolation and following centrifugation, we added it in a 1:1 ratio to the supernatant to obtain a precipitate.

Residual concentration of SDS after PHA isolation procedure and also the efficiency of KDS precipitation from SDS solutions with different initial amounts of the detergent were determined employing a spectroscopic assay based on the use of Stains-All dye (Sigma-Aldrich, Darmstadt, Germany) as described by Rupprech et al. [29]. The analysis was performed using a microplate reader (ELISA reader, ELx808, BioTek, Winooski, VT, USA).

## 3. Results and Discussion

Every isolation experiment was preceded by the cultivation of microorganisms *H. halophila* and *S. thermodepolymerans* in mineral media with the most suitable carbon sources (glucose for *H. halophila* and xylose for *S. thermodepolymerans*) to obtain biomass with high PHA content. Within individual cultivations, we tried to keep the cultures under the same conditions for gaining results of biomass and PHA content as similar as possible, despite we measured both characteristics (biomass content gravimetrically and PHA content by GD-FID) within each experiment for independent comparison among results of cultivations. For each experiment, amounts of PHA in biomass are stated under almost all tables for the comparison with the purity of isolated polymer using different treatments, because for individual experiments all the yields were related to individual cultivation results. Based on the data from experiments, for *H. halophila* average concentration of biomass was (5.5 ± 0.3) g/L with a PHA fraction in biomass of about (78 ± 7) wt.%. For *S. thermodepolymerans* average biomass concentration was (6.1 ± 0.1) g/L with (70 ± 4) wt.% PHA.

### 3.1. Optimization of SDS Concentration

Within the whole PHA isolation development process, the first step was focused on the optimal concentration of detergent SDS. The idea was to expose the extremophilic bacterial cells naturally containing compatible solutes as a part of their adaptation strategy to hypotonic conditions with low amounts of SDS to improve cell disruption and enable solubilization of hydrophobic cell components which would otherwise get attached to PHA granules reducing the purity of the materials. For both used strains, tested SDS concentrations were 1; 2.5; 5 and 10 g/L; results for individual strains are listed in Table 1. For *H. halophila* the process was carried out in a water bath during a 2 h lasting process at 70 °C; for *S. thermodepolymerans*, the process lasting the same time was carried out at 90 °C.

In the case of *H. halophila*, the most effective SDS concentration for isolation of PHA was 5 g/L, when the purity of the polymer was more than 97 wt.% together with the highest product yield considering purity and also amount of the isolated material. For this moderately halophilic strain with optimal salt concentration for growth 66 g/L, utilization of 5 g/L of SDS induces effective hypotonic cell lysis and results in high purity of the isolated material. Therefore, the most suitable concentration of SDS was used for all the following experiments employing the halophilic strain.

For *S. thermodepolymerans*, the highest purity was determined for a SDS concentration of 2.5 g/L. In comparison with the halophilic culture, the concentration is 2 times less, which might be caused by the lower content of the compatible solutes in the cells of thermophilic culture. It can be expected that thermophilic bacteria are less prone to hypotonic lysis as compared to halophiles. This expectation is also supported by the fact that purities of PHA materials obtained for thermophilic *S. thermodepolymerans* are substantially lower than those obtained in its halophilic counterpart *H. halophila*. Further, aside from the purity of the material, we have also considered the yield of the isolation process which was highest for 5 g/L of SDS leading to the establishment of the most suitable concentration of SDS of 5 g/L—the same as for *H. halophila*.

The team of Arikawa et al. (2017) focused on using SDS in combination with sonication for the isolation of PHA from biomass of the mesophilic bacterium *Cupriavidus necator*, when the optimal concentration of detergent was set on 33 g/L [30]. In our case, we used a more than 6-times lower amount of detergent due to the advantageous effect of hypotonic shock, which is applicable on strains producing compatible solutes (for instance halophiles and thermophiles). Further, our approach relying on hypotonic shock does not require any special equipment, on the contrary, sonification of the bacterial cells can be challenging especially in scales of industrial production of PHA.

### 3.2. Optimization of “Heat Treatment”

Subsequently, the second step in the isolation process development was to optimize temperature and time of isolation; together, this process can be called “heat treatment”. Within the first experiment focused on SDS concentration, “heat treatment” was represented by the application of a temperature of 70 °C for 2 h, but despite quite high yields, using different temperature can be more effective, or lowering the temperature could be more economically suitable, eventually. Based on previous results, time of exposition was set at 2 h, strain *H. halophila* was exposed to temperature 50 °C, 70 °C (as control—for comparison see Table 1), 90 °C and combination of cooling-down at 4 °C and following heating-up at 70 °C. The optimization process for *S. thermodepolymerans* included a little different strategy, when, in contrast to the halophilic strain, a lower temperature of 30 °C was also tested (we hypothesized that even low temperature could damage cell envelope of thermophilic bacterium). Other tested temperatures were 50 °C, 70 °C, 90 °C (as control—for comparison see Table 1) and also a combination of cooling-down at 4 °C and following heating-up at 70 °C as same as for *H. halophila*. The results of the experiments are listed in Table 2.

Based on the results presented in Table 2, the most successful “heat treatment” for isolation of PHA from the halophilic strain considering the purity of isolated material was a temperature of 90 °C for 2 h, when the purity was higher than 99 wt.%. Despite a little lower purity after isolation at 70 °C, the yield of the material was higher than for the highest tested temperature 90 °C and also higher than for the combination of cooling-down and following heating-up. For subsequent isolation experiments, a treatment representing 2 h of heating at 70 °C was applied for strain *Halomonas halophila*.

The optimization process for *S. thermodepolymerans* included a little different strategy; in contrast to the halophilic strain, we tested a lower temperature (30 °C), but the effectivity of isolation was not too high (the purity was around 67 wt.% and the yield was 0.73). Based on these data, the most successful “heat treatment” was keeping the sample at 90 °C for 2 h; here, the purity exceeded 99 wt.%, hence, it was very similar to using a temperature of 70 °C for *H. halophila*. The combination of cooling followed by heating was not too successful for the thermophilic strain, whereas exposition to low temperature presumably led to the development of enhanced resistance to thermal treatment as was already reported in the literature [31]. This effect was described for mesophilic bacterial strains *E. coli* [32] and *C. necator* [33] after treatment at low temperature (0–5 °C). It was proved that enhanced resistance against elevated temperature was caused by the changes in the fatty acids composition in cell membranes [31]. Despite the fact that the effect was not described for thermophilic strains in detail, it is very likely that also in this case the low-temperature treatment resulted in active modulation of cell membrane lipids, which negatively impacted the susceptibility of the bacterial cells to hypotonic lysis. For our experiment, lower efficiency of combined “heat treatment” can be observed compared with keeping the cells at 90 °C for 2 h. To conclude, the most suitable “heat treatment” procedure for the thermophilic strain *S. thermodepolymerans* was keeping the isolation mixture at 90 °C for 2 h.

### 3.3. Testing of Different Ratios SDS Solution: Biomass

After optimization of SDS concentration and “heat treatment”, the following step was focused on the influence of initial biomass amount on the purity of isolated material and the yield of the process. This is very important since PHA production is usually performed as a high cell density cultivation [34], therefore the robustness of the isolation process with respect to the amount of biomass employed per isolation batch is a very important factor determining the viability and feasibility of the process. Therefore, we tested different initial concentrations of biomass, while keeping the volume of SDS solution constant (5 g/L). Moreover, an increasing portion of biomass per isolation batch can also reduce the cost of the isolation process (lower SDS amount) and significantly decrease the environmental burden caused by the presence of SDS in wastewater.

Based on the results listed in Table 3, it is possible to state that the process is very robust regarding the initial biomass concentration; purities of material isolated from different biomass amounts were greatly similar. The same goes for almost all yields for both strains. Therefore, the developed isolation procedure can be advantageously applied on biomass obtained from high-cell-density cultivation (e.g., fed-batch cultivation).

### 3.4. Effect of Different Isolation Approaches on the Molecular Weight of the Polymer

The following experiment was focused on the determination of the molecular weight of obtained PHA materials; the main goal was to evaluate the influence of the isolation procedure on the molecular weight of the polymer.

Isolation of polymer using SDS solution (5 g/L) did not manifest in the decrease of molecular weight of PHA as can be seen in Table 4. For comparison, PHA was isolated from dried biomass using a procedure involving 12 h lasting incubation in chloroform at 70 °C in a thermoblock. To eliminate differences from sample preparation, all analyzed PHA samples were subjected to the same treatment. Despite the slight differences, values of molecular weight were in principle very similar at least for polymers isolated from *S. thermodepolymerans*. Only a little decrease can be observed which could be caused by enhanced temperature 90 °C used for isolation with SDS. In the case of *H. halophila*, isolation with SDS led to enhancement of molecular weight up to 1.5-times. It should be pointed out that the M_w_ of the polymer produced by *H. halophila* is very high as compared to other PHA producers [8] including *S. thermodepolymerans*. It can be assumed that the higher molecular weight of polymer after SDS isolation treatment might be caused by enhanced solubility of longer chains in chloroform after elimination of other cells components. 12 h lasting isolation at 70 °C in chloroform is possibly more efficient for shorter polymer chains with lower molecular weight. PDI values were very low for all studied isolation setups, indicating the high uniformity of generated biopolyesters in terms of molecular mass distribution, which is a beneficial feature for further polymer processing.

### 3.5. Determination of Material Purity by Infrared Spectroscopy

**Figure 1:**
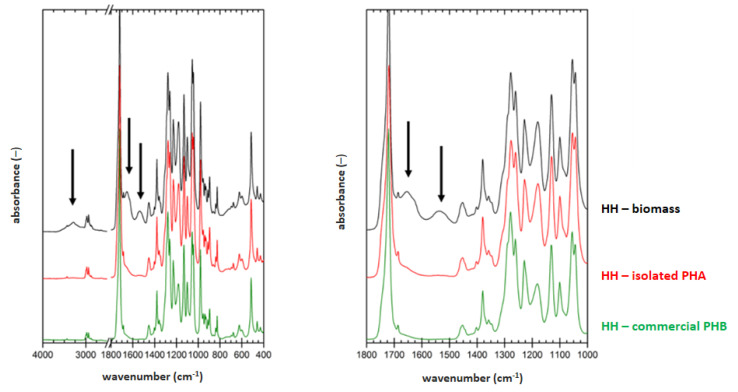
FTIR spectra of original biomass of *H. halophila* (black), polymer isolated via optimized approach (red) and commercial P(3HB) sample (Biomer) (green); arrows show absorption bands for components of biomass (3300; 1640 and 1540 cm^−1^—proteins).

**Figure 2:**
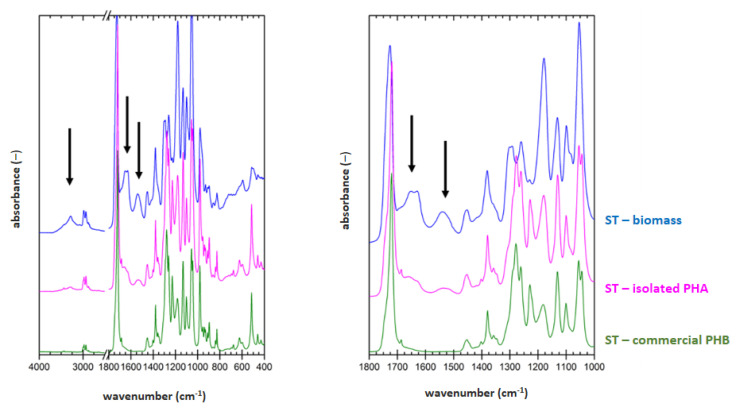
FTIR spectra of original biomass of *S. thermodepolymerans* (blue), polymer isolated via optimized approach (magenta) and commercial P(3HB) sample (Biomer) (green); arrows show absorption bands for components of biomass (3300; 1640 and 1540 cm^−1^—proteins).

FTIR technique was applied on samples of dried biomass, PHA polymer isolated by optimized process for each strain (2 h lasting treatment at 70 °C for *H. halophila* (HH) or 90 °C for *S. thermodepolymerans* (ST) in solution with 5 g/L SDS), and also for commercial P(3HB) (Biomer) as a reference material. Result are shown in Figure 1 and Figure 2.

To evaluate the purity of the polymer isolates from FTIR spectra, characteristic spectral signatures of proteins can be used. These can be found at about 3300 (referred to as amide A band), 1640 cm^−1^ (referred to as amide I band) and 1540 cm^−1^ (referred to as amide II band). All these characteristic bands are marked with arrows in the biomass spectra in Figure 1 and Figure 2. As expected, the protein signal is completely absent in spectrum of the reference P(3HB) material. In the FTIR spectra of polymer isolates obtained from both tested strains, significant decrease of the protein signal, compared to the original biomass, indicates high effectivity of the polymer purification during the extraction process. As can be seen from the comparison of Figure 1 and Figure 2, lower content of the residual biomass was found for the polymer isolated from *H. Halophila*. This is in good agreement with the results of GC-FID. Based on the gravimetric yield of the chromatographic analysis, we determined polymer purity (86.4 ± 1.4) wt.% for *S. thermodepolymerans* and (91.3 ± 1.5) wt.% for *H. halophila*.

### 3.6. Electron Microscopy Imaging

Selected samples were analyzed using electron microscopy techniques, specifically, samples of microbial cells of *H. halophila* and *S. thermodepolymerans* and isolates of PHA from both strains.

**Figure 3:**
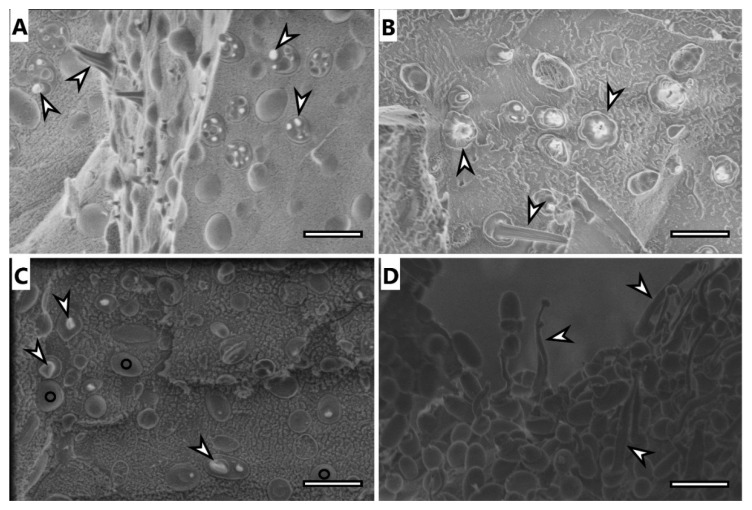
Cryo-SEM image of: (**A**) *H. halophila* biomass before extraction of PHA granules, (**B**) PHA granules isolated from *H. halophila*, (**C**) *S. thermodepolymerans* biomass before extraction of PHA granules, (**D**) PHA granules isolated from *S. thermodepolymerans*, needle deformation of PHA granules marked with arrowhead, cells with fractured cell wall revealing the surface of plasma membrane marked with O, scale bar 2 µm.

Cryo-SEM image of *H. halophila* (Figure 3A) shows rod-shaped cells containing several granules of PHA. Most of the cells were fractured during the freeze-fracturing procedure and revealed the intracellular content of PHA. As previously described [35], PHA remain elastic even at temperatures of liquid nitrogen and can be observed being pulled out of the cells, showing “needle type” deformation (arrowhead in Figure 3) or as holes in cells (concave deformation) when the granule was pulled out of the cell completely. Cells of *S. thermodepolymerans* (Figure 3C), containing 1 or 2 granules of PHA also deformed by freeze-fracturing. In the image, it is possible to observe also fracture of the cell wall revealing the surface of the plasma membrane, marked with O. The image of isolated granules from *H. halophila* (Figure 3B) shows that the granules merged, but still remained elastic. PHA isolated from *S. thermodepolymerans* (Figure 3D), however, stayed separate after the extraction as individual oval granules, which were also affected by freeze-fracturing and show needle deformation.

**Figure 4:**
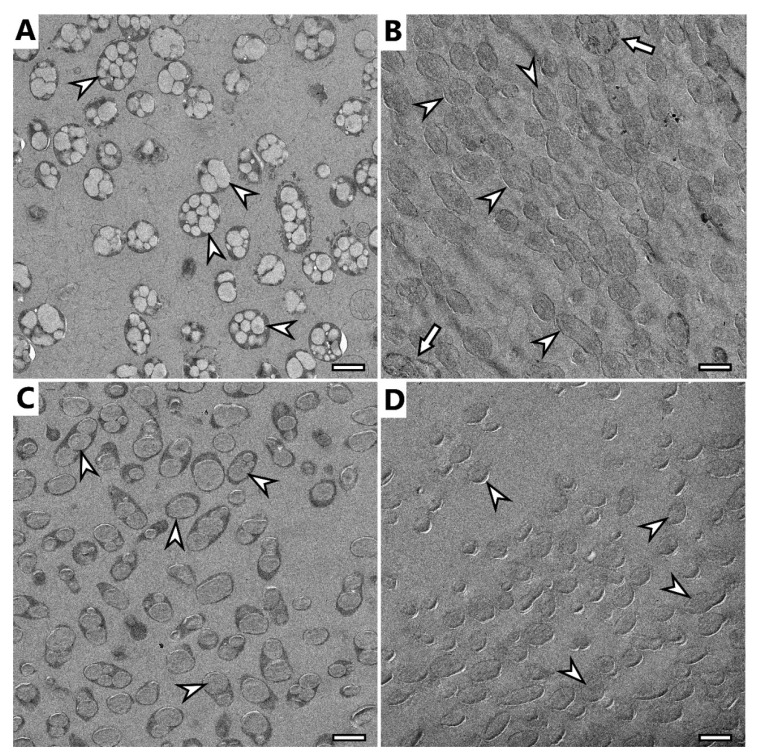
TEM image of (**A**) *H. halophila* biomass before extraction of PHA granules, (**B**) PHA granules isolated from *H. halophila*, (**C**) *S. thermodepolymerans* biomass before extraction of PHA granules, (**D**) PHA granules isolated from *S. thermodepolymerans*, PHA granules marked with arrowhead, intact cells after extraction marked with arrow, scale bar 1 µm.

TEM observation confirmed previous findings of cryo-SEM. *H. halophila* contained several smaller granules in their cells (Figure 4A), while *S. thermodepolymerans* contained 1–3 larger granules. Generally, it is described that larger PHA granules are beneficial for the recovery process [36], which makes *S. thermodepolymerans* of even higher interest for an eventual industrial-scale application. Images of isolated granules also confirm the shape observed in cryo-SEM: isolates from *H. halophile* merged into irregularly shaped granules, while granules from *S. thermodepolymerans* of regular oval shape stayed separate. In Figure 4B,D, it is possible to see shades surrounding the granules. Based on the results of purification, the shades could be caused by contamination originating from disrupted cellular fragments, or by PHA granule membrane remnants. It is also possible to observe a few intact cells (Figure 4B marked with arrow), thus confirming the results of the determination of purity of the isolates—for instance (91.3 ± 1.5) wt.% of PHA for *H. halophila* using standard optimized process.

### 3.7. Wastewater Management—Precipitation of KDS from SDS after Isolation

As same as other detergents, also SDS serves as a contaminant of wastewater with possible harmful effects on wastewater management. Therefore, we focused on the removal of the irritant compound SDS from the supernatant after the isolation process. We have tested several approaches (data not shown), and the most successful procedure was simple and feasible precipitation of SDS in the form of poorly water-soluble potassium dodecyl-sulfate (KDS) in the presence of 2 M KCl. The efficiency of the SDS removal process was determined via measurement of SDS concentration using the protocol of Rupprech et al. (2015) [29]. Data are demonstrated in Table 5.

**Figure 5:**
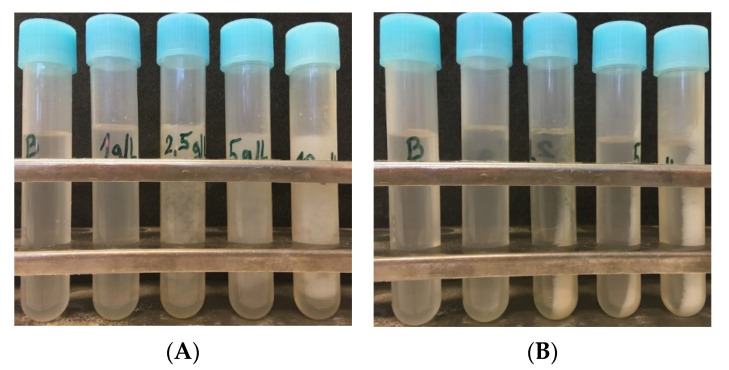
KDS precipitate using 2 M KCl after isolation with different SDS concentrations—from the left: blank, 1 g/L, 2.5 g/L, 5 g/L, 10 g/L: (**A**) before centrifugation; (**B**) after centrifugation.

Based on presented data, it seems that removal of SDS from a wastewater stream by simple addition of non-toxic and cheap KCl is a very effective process since after this treatment the residual concentration of SDS in the supernatant after isolation was not detectable for almost all the samples. The formation of KDS precipitate is a very quick and straightforward method to remove SDS used for isolation of PHA granules, since the formation of the precipitate can be observed immediately after the addition of KCl into supernatants after isolation as shown in Figure 5.

A different strategy to get rid of the detergent after isolation was used by the team of Samorì et al. (2015), who isolated PHA polymer from bacterial biomass. For this purpose, they used ammonium laurate (most efficient at a concentration of 200 wt.%), which acts similarly to SDS in terms of microbial cell disruption and solubilization of hydrophobic contaminants. After separation of the polymer by centrifugation, the addition of CO_2_ into supernatant leads to a decrease of the pH-value, and switches the detergent into poorly water soluble neutral (protonated) lauric acid with the possibility of separation by centrifugation; resulting water phase containing ammonium hydrogen carbonate can serve as a nitrogen source for microorganisms [37]. Our approach relies on the application of the lower amount of inexpensive and abundant detergent SDS, which can be simply removed from wastewater by precipitation. It should be pointed out that unlike for ammonium laurate, the removal of the SDS from the supernatant in form of KCl occurs in a non-destructive way, and it is very likely that SDS could be regenerated for instance by ultradialysis in excess of Na^+^ ions. Hence, our concept enables not only removal of detergent from wastewater, but also holds a promise of SDS recovery, which would be very beneficial with respect to economic aspects of the PHA isolation process. Nevertheless, the possibility of SDS recovery from KDS precipitate deserves further investigation, which is out of the scope of this preliminary work.

## 4. Conclusions

In this work, we developed a procedure for the isolation of PHA materials from extremophilic microbial cells. The method is based on the exposition of the bacterial cells naturally containing high intracellular concentrations of compatible solutes as part of their adaptation strategy to extreme hypotonic conditions induced by the diluted solution of SDS (optimal concentration is about 5 g/L of SDS) at elevated temperature. Our results indicate that such conditions lead to disruption of the cells and release of PHA granules. Moreover, SDS apart from its cell-disruptive function also solubilizes hydrophobic cell components which would otherwise get attached as contaminants to PHA materials. The procedure seems to be simple, robust, and feasible in industrail condtions. The purity of obtained materials (usually above 95%), as well as yields of the procedure (usually about 0.9), reach high values. If even higher purity of the material is needed, additional steps such as the washing of the materials with proper organic solvents or other agents can be involved. Furthermore, since leftovers of the detergent SDS in process wastewater might dramatically decrease the positive ecological features of the process, we also focused on the removal of SDS. We have developed a simple, cheap, and safe technique that is based on the precipitation of SDS in the presence of KCl. The resulting precipitate can be simply removed by decantation or centrifugation, hence, SDS does not contaminate the wastewater of the process. Moreover, there is also the possibility to regenerate SDS which would substantially improve the economic feasibility and overal sustainability of the process. To sum up, the developed strategy for PHA isolation is compatible with Next-Generation Industrial Biotechnology concepts, it is simple, cheap, robust, provides PHA materials in high purity and at high yields and is also ecologically friendly.

## Figures and Tables

**Table 1:** Effect of different SDS concentrations on PHA purity and yield during 2 h lasting isolation at 70 °C for *H. halophila* and 90 °C for *S. thermodepolymerans*.

Strain	SDS (g/L)	PHA Purity (wt.%)	Yield (−) ^1^
*H. halophila*	1	88.0 ± 0.4	0.99
	2.5	91.4 ± 2.7	0.99
	5	97.1 ± 3.7	1.11
	10	89.6 ± 1.4	1.09
*S. thermodepolymerans*	1	65.7 ± 0.8	0.95
	2.5	87.7 ± 3.6	0.97
	5	86.4 ± 1.4	1.00
	10	85.5 ± 0.4	0.92

^1^ Yield is defined as PHA (g/L) for individual isolation way divided by PHA (g/L) obtained from biomass without previous isolation step. PHA content in the biomass: *H. halophila* (84.02 ± 0.61) wt.%, *S. thermodepolymerans* (64.43 ± 5.96) wt.%.

**Table 2:** Effect of different “heat treatments” on PHA purity and yield during isolation in 5 g/L SDS solution.

Strain	“Heat Treatment”	PHA Purity (wt.%)	Yield (−)
*H. halophila*	50 °C (120 min)	97.5 ± 1.2	0.92
	70 °C (120 min)	96.7 ± 0.3	0.97
	90 °C (120 min)	99.7 ± 0.1	0.90
	4 °C (120 min) + 70 °C (120 min)	94.3 ± 1.5	0.92
*S. thermodepolymerans*	30 °C (120 min)	67.6 ± 0.8	0.73
	50 °C (120 min)	81.4 ± 4.3	0.78
	70 °C (120 min)	96.7 ± 2.4	0.79
	90 °C (120 min)	99.8 ± 2.8	0.91
	4 °C (120 min) + 90 °C (120 min)	86.2 ± 2.1	0.87

PHA content in the biomass: *H. halophila* (82.27 ± 0.09) wt.%, *S. thermodepolymerans* (69.57 ± 0.79) wt.%.

**Table 3:** Effect of different biomass content on PHA purity and yield applied during 2 h lasting isolation in 5 g/L SDS solution at 70 °C for *H. halophila* and 90 °C for *S. thermodepolymerans*.

Strain	Biomass Amount in SDS Solution (g/L)	PHA Purity (wt.%)	Yield (−)
*H. halophila*	1.83	96.2 ± 1.9	0.86
	4.21 (standard process)	96.6 ± 0.3	0.99
	8.40	95.9 ± 7.1	1.04
	11.86	91.5	0.96
	17.72	94.9	1.03
	22.65	95.9 ± 1.0	1.06
*S. thermodepolymerans*	1.91	96.3 ± 1.3	0.89
	3.76 (standard process)	101.1 ± 4.7	0.92
	8.88	93.4 ± 0.5	0.77
	11.72	84.1	0.80
	16.01	93.3 ± 2.4	1.01
	20.99	94.1	0.94

PHA content in the biomass: *H. halophila* (75.5 ± 0.1) wt.%, *S. thermodepolymerans* (68.5 ± 0.0) wt.%.

**Table 4:** The molecular weight of isolated PHA polymer.

Strain	“Treatment”	M_w_ (kDa)	PDI ^2^ (−)
*H. halophila*	PHA isolated from dried biomass	1050.4 ± 1.8	1.20 ± 0.04
	70 °C (120 min)—standard process	1581.9 ± 5.3	1.46 ± 0.19
*S. thermodepolymerans*	PHA isolated from dried biomass	711.5 ± 1.6	1.17 ± 0.01
	90 °C (120 min)—standard process	686.4 ± 16.1	1.23 ± 0.03

^2^ PDI (polydispersity index) is defined as M_w_/M_n_. M_w_: weight average molecular mass; M_n_: number average molecular mass.

**Table 5:** Results of SDS determination before and after KDS precipitation.

Parameter	SDS (g/L)			
Concentration used for isolation	1	2.5	5	10
In supernatant after isolation	0.6	1.8	4.9	10.7
Residual SDS concentration after KDS precipitation	0.02	n.d. ^3^	n.d.	n.d.
KDS yield (mg)	26.8	64.9	169.9	316.0

^3^ n.d.—no detectable.
